# The Anatomical Society's core anatomy syllabus for undergraduate nursing

**DOI:** 10.1111/joa.12782

**Published:** 2018-02-07

**Authors:** S. A. Connolly, T. H. Gillingwater, C. Chandler, A. W. Grant, J. Greig, M. Meskell, M. T. Ross, C. F. Smith, A. F. Wood, G. M. Finn

**Affiliations:** ^1^ Department of Anatomy Edinburgh Medical School: Biomedical Sciences College of Medicine and Veterinary Medicine University of Edinburgh Edinburgh UK; ^2^ Department of Nursing, Health and Social Science University of Edinburgh Edinburgh UK; ^3^ School of Life Sciences Glasgow Caledonia University Glasgow UK; ^4^ School of Health and Social Care Edinburgh Napier University Edinburgh UK; ^5^ School of Nursing, Midwifery & Health Systems Health Sciences Centre University College Dublin Dublin Ireland; ^6^ Centre for Clinical and Medical Education University of Edinburgh Edinburgh UK; ^7^ Brighton & Sussex Medical School University of Sussex Brighton UK; ^8^ Health Professions Education Unit Hull York Medical School University of York York UK

**Keywords:** anatomy, core syllabus, curriculum, education, human, integrated learning, learning outcomes, nursing, pre‐registration, syllabus, undergraduate, United Kingdom

## Abstract

The Anatomical Society has developed a series of learning outcomes in consultation with nursing educators delivering anatomical content to undergraduate (preregistration) nursing students. A Delphi panel methodology was adopted to select experts within the field that would recommend core anatomical content in undergraduate nursing programmes throughout the UK. Using the Anatomical Society's Core Gross Anatomy Syllabus for Medical Students as a foundation, a modified Delphi technique was used to develop discipline‐specific outcomes to nursing graduates. The Delphi panel consisted of 48 individuals (*n* = 48) with a minimum of 3 years' experience teaching anatomy to nursing students, representing a broad spectrum of UK Higher Education Institutions. The output from this study was 64 nursing specific learning outcomes in anatomy that are applicable to all undergraduate (preregistration) programmes in the UK. The new core anatomy syllabus for Undergraduate Nursing offers a basic anatomical framework upon which nurse educators, clinical mentors and nursing students can underpin their clinical practice and knowledge. The learning outcomes presented may be used to develop anatomy teaching within an integrated nursing curriculum.

## Introduction

The World Health Organization defines nursing as: ‘encompassing autonomous and collaborative care of individuals of all ages, families, groups and communities, sick or well and in all settings. It includes the promotion of health, the prevention of illness, and the care of ill, disabled and dying people’ (WHO Europe 2009: p. 5).

There are four main divisions of nursing taught at undergraduate level in the UK: Adult nursing – the care of people aged 18 or over; Children's nursing – the care of children and young people from birth to late adolescence; Learning disabilities nursing – the care of people of all ages who have learning disabilities; Mental health nursing – the care of people of all ages who have mental health problems (NMC, 2010: p. 147).

Despite the existence of these distinct branches of nursing, a knowledge, understanding and application of human anatomy alongside other basic sciences, collectively known as ‘biosciences’, falls under the remit of generic competency which all undergraduate nurses must be able to demonstrate by the end of the bachelor's degree programme. (NMC, 2010: pp. 95 and 147).

The biosciences required by nurses are anatomy, physiology, biochemistry, pathology, pathophysiology, genetics, microbiology, pharmacology and biophysics. A working knowledge of these areas is deemed to be required for safe and competent practitioners (WHO Europe, 2009; NMC, 2010).

With regard to anatomical knowledge and understanding, at present, the guidance by the UK Nursing and Midwifery Council (NMC) to academics teaching nurses within accredited nursing programmes simply states that a knowledge, understanding and application of human anatomy is essential to ensure eligibility to register to practise upon graduation (NMC, 2010). Despite anatomy being deemed a fundamental requirement for safe clinical practice, there is evidence since 1980 documenting a widespread ‘bioscience problem’ affecting anatomy and other elements of nursing education, both nationally (Nicoll & Butler, 1996; Jordon et al. 1999; Clancy et al. 2000; Davies, 2010; Smales, 2010; Andrew et al. 2015; Taylor et al. 2015) and internationally (Akinsanya & Hayward, 1980; Friedel & Treagust, 2005; Craft et al. 2013).

When the studies were cross‐matched and compared, they revealed common and consistent findings in student anxiety and low self‐confidence in learning and applying bioscientific knowledge, due to a suspected lack of conceptualisation. Anecdotal reports from students and teaching staff have attributed these issues to a lack of sufficient learning and teaching time for bioscience subjects, including anatomy (Jordon et al. 1999; McVicar et al. 2015; Taylor et al. 2015). This may be further complicated by a lack of confidence displayed by many clinical mentors with regard to integrating bioscientific principles into clinical practice (Molesworth & Lewitt, 2015). Despite this, nursing students have highlighted a fundamental knowledge in anatomy as an important element of their practice (Meskell & O'Connor, 2007).

Several previous studies have attempted to address deficits in bioscience education by implementing and investigating various paedagogical approaches (Davies et al. 2000; Green et al. 2000, 2006; Al‐Modhefer & Roe, 2010; Koch et al. 2010; Efstathious & Bailey, 2012; Craft et al. 2016; Montayre & Sparks, 2017), whereas others have focused on natural science entry requirements as key performance indicators (McKee, 2002; Van Rooyen et al. 2006). However, the existing body of evidence is largely based on data gathered across only one or two sites per study, compromising generalisability and maximising the likely impact of local influencing factors (Andrew et al. 2015). This has led to the conclusion that student's' difficulty in learning and assimilating biosciences is due, at least in part, to a lack of explicit guidelines from the NMC, thereby creating widespread geographical variability in curricular content and delivery (McVicar et al. 2015; Taylor et al. 2015).

In response to the need for enhanced integration and to address the bioscience issue, the Bioscience in Nursing Education group (BiNE) was established by the Higher Education Academy (HEA) to create a quality assurance framework for bioscience education in nursing. The BiNE (2016) framework incorporates a list of broad learning objectives spanning anatomy, physiology, biology, pathophysiology, pharmacology and genetics. Although this framework has provided additional clarity, the term ‘Bioscience’ remains broad, leading to issues with identifying and resolving problems specific to scientific domains.

To counter the lack of subject‐specific guidelines, particularly with respect to anatomy, we sought to develop the first national advisory core anatomy syllabus for undergraduate nurses. Subject‐specific learning outcomes have been shown to be effective for curriculum design, teaching and learning, and developing assessment. Additionally, anatomy‐specific outcomes can assist greatly in the systematic design of integrated modules and programmes (Biggs & Tang, 2007; Kennedy et al. 2009).

The NMC and the General Medical Council (GMC, 2009) endorse the development of transparent educational standards that can provide clarity on areas where educational integration can be facilitated across health and social care to reflect the integrated, multiprofessional dynamic in modern healthcare service delivery (NHS Scotland 2007, NMC, 2017, NHS Scotland 2014–15). This is also supported by disciplines allied to health education as exemplified by the Anatomical Society and International Association of Anatomists creation of advisory core syllabi to provide a high‐quality ‘common‐sense’ approach to anatomical teaching and learning (McHanwell et al. 2007; Smith et al. 2016a,b; Moxham et al. 2017).

The Anatomical Society has syllabi projects in medicine, dentistry and pharmacy, and has engaged with stakeholders in each respective field to produce explicit advisory guidance on anatomy‐learning outcomes. The rationale for developing discipline‐specific core anatomy syllabi is a response to the reduction of anatomical teaching hours across all healthcare disciplines and to provide transparent and detailed guidance on the level of anatomical content expected at the point of registration. The outcomes presented here for nursing have been developed by life science nurse educators, with consensus analysis facilitated by the Anatomical Society, thereby aiming to minimise variability by providing a coherent content guide to assist anatomical teaching and learning.

The recommended core anatomy presented below consists of a list of defined learning outcomes, arranged by systems. Table 1 provides contextual information to guide curriculum planners and teachers with the implementation and integration of the syllabus outcomes into the curriculum in a number of ways, outlined below. The list is illustrative as opposed to exhaustive. Additionally, the contextual information will assist nurse educators to signpost the clinical relevance of the anatomy to students. It can also be used by clinical mentors when integrating theoretical knowledge into practice to provide clinical context. Finally, it serves to aid mapping of learning outcomes and assessment throughout the preregistration nursing curriculum.

**Table 1:** Contextual information to support the integration of outcomes into the curriculum

Learning outcome	Clinical context
Anatomical terminology	
1	Position and relationship of anatomical structures. To describe patient positioning. Descriptors for the location of injury and pain. Keeping accurate records and interpreting documentation from other healthcare team disciplines
2	Joint movement and related injuries
3	Assessment and monitoring of normal and compromised movement: musculoskeletal injuries, rheumatic disease, post‐procedural/operative care of arthroscopy, orthopaedic and spinal. Impact on activity of daily living assessment. Pain assessment
4	Normal development vs developmental anomalies Effects of adverse health behaviours on anatomical structures throughout the lifespan, e.g. smoking, excessive alcohol intake Systems based structural geriontological considerations correlated to pathology, physiological homeostatic adaptation and pathophysiology, i.e. vasculature changes, loss of bone density, detrusor muscle instability, etc. Drug dosage adjustments and modified calculations Choice of medication administration route in different patient populations
5	Care of pressure areas and prevention of pressure ulcers. Assessment of pressure ulcer grade 1–4. Landmarks for intramuscular injection (IM), i.e. humeral head for deltoid injection and the anterior superior iliac spine for gluteus medius injection
Histological overview	
6	Homeostatic mechanisms, pathophysiology and pharmacological interventions
7	Physiological: endocrine secretions, bladder and uterus expansion, protection from abrasion, absorption and transport. Clinical: mucosal membrane assessment, oral lesions, carcinoma, granulation in wound care
8	Anatomy. Physiology. Pathology. Pathophysiology. Pharmacological action
9	Support, protection and mobility. Cartilage injuries and healing
10	Types of contraction, i.e. voluntary/involuntary
11	Supportive and protective role in relation to gross structures, extracellular fluid spaces, blood, immunity. Autoimmune disorders, i.e. lupus erythematous
Nervous system	
12	Vital sign regulation, pain, reflexes, sensory and motor function throughout the body. Somatic sensory pathology, i.e. shingles, motor neuron disease, multiple sclerosis
13	Vital sign ‘control centres’, i.e. temperature regulation (hypothalamus), baroreceptors (pons) and chemoreceptors (medulla oblongata), pituitary tumours, schizophrenia (limbic system)
14	Parkinson's disease, aminergic and cholinergic systems and related pharmacology, gait, balance, coordination, muscle tone (i.e. hypotonia)
15	Relate to special senses Neurodegeneration, e.g. dementia Effects of stroke on various brain regions, e.g. dysarthria. Neuropsychiatric disorders and intellectual disability
16	Relate to corresponding regions of the brain, and assessment and interventions for activities of daily living
17	Blood‐brain barrier, meningitis, subdural and extradural haemorrhage/haematoma
18	Anosmia (lack of smell affects appetite); blindness; diplopia (double vision); trigeminal neuralgia (facial pain and spasm); Bell's palsy; hearing loss; tinnitus; dizziness; dysphagia. Cranial nerve abnormalities may be indicative of cervical spine and/or brainstem pathology/injury
19	Hydrocephalus, raised intracranial pressure related to consciousness
20	Infection, i.e. otitis media, labyrinthitis, dizziness and loss of balance. Administrating ear drops
21	Conjunctivitis, cataracts, glaucoma, red eye, administering eye drops, basic eye care, Glasgow Coma Scale (GCS) pupil assessment
22	Reflex and pain, post‐care and education of a lumbar puncture, cauda equina
Musculoskeletal system	
23	Normal and abnormal integrity, fractures, osteoporosis
24	Bone marrow, immunity, pre‐ and post‐care of bone marrow aspirate and stem cell transplants
25	Mobility, moving and handling, sprains, arthritis, tendonitis, bursitis, nerve impingement, rotator cuff injury, i.e. frozen shoulder, carpel tunnel syndrome
26	Neurovascular monitoring, myalgia, compartment syndrome, administration of intramuscular injection/safe sites, assessment of mobility, gait, muscle tone and general actions that impact on functional activities such as sitting, standing, lying, lifting and exercising. Rehabilitation, multidisciplinary communication, interpreting and supporting physiotherapy instructions, falls prevention
27	Quadriplegia, paraplegia, lordosis, kyphosis, scoliosis, moving and handling, pressure ulcer care
Integumentary system	
28	Cellulitis, burns, inflammation, decreased turgor in dehydration, skincare, assessment and medical referral of suspicious moles for skin cancer, topical medication
29	Anaesthetic action, referred pain, i.e. cholecystitis pain referred to the left shoulder via the phrenic nerve at level C3/4/5 due to diaphragmatic irritation. Monitoring diminished sensation postoperatively, i.e. patient‐controlled analgesia (PCA) *in situ*, paraesthesia
Cardiovascular system	
30	Palpation of apex heartbeat, electrocardiogram (ECG) electrode placement
31	Normal heart rhythm, blood pressure, cardiac output, arrhythmias, pericarditis, pericardial effusion, cardiac tamponade, care of pacemakers, heart failure, patent foramen ovale, septal defects, ECG, cardiac monitoring, cardioversion
32	Mechanism of circulation, rheumatic fever, endocarditis, cardiac valve insufficiencies, i.e. mitral valve regurgitation, aortic/pulmonary stenosis
33	Ischaemic heart disease, i.e. myocardial infarction, stable/unstable angina. Pre‐ and postoperative angiogram/percutaneous coronary intervention (PCI) pre‐, intra‐ and post‐care
34	Circulatory function, i.e. oxygenation, deoxygenation and diffusion
35	Circulatory function in chronic obstructive pulmonary disease (COPD) and right‐sided heart failure, pulmonary oedema and effusion, pulmonary embolism
36	Atherosclerosis, visceral ischaemia/infarct, aortic aneurysm, thrombus and embolism, peripheral venous disease (PVD) and peripheral arterial disease (PAD) – compression bandaging and care of PVD/PAD ulceration. Blood pressure, profusion (i.e. pallor, central and peripheral cyanosis/mottling), wound healing
37	Intravenous drug administration, cannulation, phlebotomy, phlebitis, varicose veins, deep vein thrombosis
38	Stroke, headache, haemorrhage, care of central lines
39	Portal hypertension
Respiratory system	
40	COPD, acute respiratory distress syndrome, asthma, pneumothorax, flail chest
41	Airway management, epistaxis, laryngitis, pharyngitis, tracheostomy care, airway suction
42	Normal and abnormal respiration, i.e. tachypnoea, hypoventilation, upper and lower respiratory chest infections, i.e. pneumonia Pleurisy pre‐, intra‐ and postoperative care of lobectomy Administration of oxygen/nebulisers and/or inhalers Peripheral oxygen monitoring (aka SpO_2_ monitoring)
43	Unstable cervical spine trauma at C3/4/5, quadriplegia, phrenic nerve palsy, assessment of bilateral chest movement
Gastrointestinal system	
44	Oral lesions, candida, sublingual drug administration, oral hygiene
45	Bolus formation, xerostomia, mumps, autoimmune disorders that affect salivary secretions, i.e. HIV
46	Swallowing, aspiration, nasogastric (NG) insertion, feeding and aftercare
47	Peritonitis, postoperative adhesions and abdominal pain, abdominal and inguinal hernia
48	Digestion, absorption, NG placement, ulceration (haematemesis), gastric dumping
49	Peristalsis, gastro‐oesophageal reflex disease (GORD), referred epigastric pain, the composition and flow of bile and pancreatic juices and faecal incontinence
50	Bowel obstruction, resection and stoma care, Crohn's disease, ulcerative colitis, colorectal abscess
51	Nutritional and drug absorption, stool colour and formation, constipation/diarrhoea, excretion
52	Hepatitis, cirrhosis, jaundice, cholecystitis, cholelithiasis, pancreatitis, drug and alcohol metabolism, overdoses, liver failure
53	Abdominal and/or pain assessment, record keeping and multidisciplinary communication
Urinary system	
54	Urinary catheterisation, urinary tract infections
55	Vascularisation of the kidney, nephrectomy, polycystic kidney disease, flank pain
56	Creation of urine and metabolic properties for urinalysis, blood pressure regulation, drug metabolism, i.e. diuretics. Acute and chronic kidney injury, dialysis
57	Urinary frequency/retention. Prostate hyperplasia/cancer
Reproductive	
58	Sexual and reproductive health and disease. Can also be used to distinguish differences between sex and gender in the broader curriculum
59	Continence and incontinence, urinary retention, rectal and vaginal prolapse, sexual function and dysfunction. Kegel exercises postpartum
60	Lactation, breast cancer, pre‐ and postoperative care of breast biopsy, mastectomy, breast reconstruction
61	Normal and abnormal stressors and associated homeostatic changes
Lymphatic system	
62	Lymphoedema, primary and secondary malignancy
63	Immunity and infection – related to microbiology and pharmacology
Endocrine	
64	Neurotransmitter and hormonal pathophysiology

### Ethical approval

The study was granted ethical approval from the University of Edinburgh Health and Social Science ethical committee from the school of nursing (Reference No: NURS021).

## Method

The established Delphi process used for the formation of the Anatomical Society's Core Regional Anatomy Syllabus for Undergraduate Medicine (McHanwell et al. 2007; Moxham et al. 2014; Smith et al. 2016a) was adopted for this study. The Delphi process is a consensus method that systematically facilitates and structures communication between experts to ascertain collective agreement on a single issue, through reiterated survey rounds (Keeney et al. 2011; Ab Latif et al. 2016; Smith et al. 2016b). The Delphi process outlined in Smith et al. (2016b) was modified to include a pilot panel of nurse educators, anatomists and clinical educational experts (*n* = 9). The role of the pilot panel was to screen the Anatomical Society's Core Regional Anatomy Syllabus for Undergraduate Medicine (Smith et al. 2016a,b) for applicability to nurses, based upon the recommendations within the BiNE (2016) framework. BiNE advised that nursing programmes undertake a system‐based approach to designing and delivering anatomical content. To ensure that the format and phraseology of the syllabus was pertinent to a wider nurse educator audience, the pilot panel were asked to use the ‘accept’, ‘reject’, ‘modify’ and/or ‘comment’ structure on each intended learning outcome to reflect the design of the online survey, prior to national circulation of the learning outcomes.

A UCAS search identified 76 higher education institutions (HEI) that delivered accredited nursing programmes. A purposive sample of life science lecturers, senior lecturers, professors, associate professors and deans with a minimum of 3 years of anatomy teaching experience in undergraduate nursing programmes were then invited to participate by email, along with a participant information sheet and consent form. Participants were invited to take part over two rounds. In phase one, participants were asked to ‘accept’, ‘reject’ or ‘modify’ with open comments to refine the intended learning outcomes. In phase two, a final consensus was sought, with options to ‘accept’ or ‘reject’ with typographic changes only. In all, 69 of the 76 HEIs were contacted to take part. Pilot panellist institutions (*n* = 4) were excluded from undertaking the online survey. Five had no staff directory on their websites and did not respond to telephone calls. Of the 69 HEIs contacted, 48 individuals from 35 HEIs took part in the online survey. There was a total of 57 panellists, including pilot phase experts and national participants from 38 HEIs across the UK: one professor, one associate professor, one associate dean, 41 senior lecturers, six teaching fellows and seven registered nurses. Of survey participants, 68.09% reported more than 10 years of experience teaching anatomy to undergraduate nursing students. The panellists agreed on the presented final core syllabus in anatomy for undergraduate nursing students.

## The syllabus

### Anatomical terminology

1. Define the following terms relative to the anatomical position: Anterior/ventral, posterior/dorsal, superior, inferior, medial, median, lateral, proximal, distal, superficial, deep, prone, supine, palmar & plantar.
2. Describe the following anatomical planes: Axial/transverse/horizontal, sagittal/vertical plane and the coronal/frontal plane.
3. Define and demonstrate the terms used to describe movement: Flexion, extension, abduction, adduction, medial rotation, lateral rotation, inversion, eversion, supination, pronation, plantar‐flexion, dorsi‐flexion, and circumduction.
4. Compare and contrast the systematic changes associated with growth and ageing in children, adults and the elderly.
5. Identify the major surface and bony landmarks in each body region (e.g. occipital protuberance, orbital ridge, nasal bones, mastoid process, cervical to sacrococcygeal vertebrae and associated joints, shoulder girdle and upper limb, sternal region, ribs and costal margin, pelvic girdle and lower limb).

### Histological overview

- Identify and describe the components of a basic cell.
- Identify and describe the features of epithelial tissues (simple squamous, stratified squamous, transitional, cuboidal, columnar and ciliated).
- Identify and describe the general structure of a neuron.
- Describe and contrast different types of cartilage (hyaline, fibrocartilage and elastic cartilage).
- Compare and contrast the structural features of skeletal, smooth and cardiac muscle.
- Describe the role of connective tissues.

### Nervous system and special senses

- Define and describe the major divisions of the central nervous system (CNS), peripheral nervous system (PNS) and autonomic nervous system (ANS).
- Identify the major divisions of the brain: The forebrain (cerebral hemispheres), the midbrain (amygdala, thalamus, hypothalamus, hippocampus, pituitary gland, pineal gland and crus cerebri), the brainstem/hindbrain (pons, medulla oblongata and cerebellum).
- Describe the difference between grey matter (e.g. nuclei, cortex and basal nuclei/ganglia) and white matter (association, commissural and projection fibres, and the corpus callosum).
- Identify the position of the frontal, parietal, temporal and occipital lobes and the major sulci/landmarks that separate them.
- Identify and briefly describe the cerebral cortex in relation to its functions, namely: motor, sensory, visual, auditory, speech; memory and emotion, decision making, social behaviour.
- Describe the structural differences between the three layers of meninges (dura, arachnoid and pia) and their relationship to the brain and spinal cord.
- Name the 12 cranial nerves and summarise their major functions in relation to PNS innervation.
- Describe the ventricular system and the formation, circulation, drainage and role of cerebrospinal fluid.
- Describe the general organisation of the outer, middle and inner ear.
- Describe the structure of the eye, eyelid, conjunctiva and lacrimal gland. Explain their importance for the maintenance of corneal integrity.
- Describe the structure of the spinal cord, a typical spinal nerve and a reflex arc, and its relation to the vertebral column.

### Musculoskeletal system

- Identify the major bones that make up the axial and appendicular skeleton and summarise their main differences.
- Outline the main differences between compact and cancellous bone.
- Describe and contrast different types of joints (synovial, fibrous and cartilaginous) and their associated structures (cartilage, tendons, ligaments, bursa) in relation to movement and stability.
- Name and describe the major muscles groups of the head, neck, thorax, abdomen, pelvis, upper limb and lower limb.
- Identify the main curvatures and features of the vertebral column, individual vertebrae (cervical, thoracic, lumbar, sacral and coccygeal) and intervertebral joints.

### Integumentary system

- Describe the epidermis, dermis & subcutaneous layers of the skin and appendages (hair follicles, sweat glands, nails).
- Summarise the contribution of the dermatomes in sensory perception and referred pain.

### Cardiovascular system

- Identify and describe the position of the heart in the mediastinum relative to the associated structures.
- Describe the four chambers of the heart (external and internal features), its specialised conduction network, and the fibrous and serous layers of the pericardium.
- Compare and contrast the structure and location of the valves of the heart.
- Describe the origin, course and main branches of the left and right coronary arteries and describe their location relative to the heart and the general area of the heart that they supply.
- Distinguish the structural differences between arteries, veins and capillaries.
- Define and demonstrate the structures of the pulmonary and systematic circulation.
- Identify and describe the course and important relationships of the major arteries and veins of the trunk, with emphasis on the aorta, superior vena cava and inferior vena cava, their major branches and associated pulse points.
- Define the deep and superficial veins and outline the course of the main veins of the upper limb and lower limb.
- Identify and describe the blood supply and venous drainage of the brain and its association to the great vessels of the heart and neck.
- Identify and describe the hepatic portal‐venous system.

### Respiratory system

- Identify the associated joints and muscles of respiration (accessory and intercostal muscles and thoracic joints, i.e. components of the sternum, ribs and costal cartilage articulations) and examine their contribution to the mechanism of breathing.
- Identify and describe the major features of the external nose, the nasal cavity, the pharynx, the larynx and the trachea.
- Describe the major features of the diaphragm, pleural layers and the lungs (lobes and fissures of the right and left lungs; bronchi, bronchioles, alveoli; surface landmarks).
- Identify and describe the course and role of the phrenic nerve in maintaining normal breathing.

### Gastrointestinal system

- Describe the major features of the oral cavity including the teeth, tongue, soft and hard palate.
- Describe the salivary glands (parotid, submandibular and sublingual) and their relationship to the oral cavity for digestion.
- Identify and describe the structure of the oesophagus and explain the role of the epiglottis in demarcating the respiratory and digestive tracts.
- Describe the relationship of the abdominal organs to the peritoneum (parietal and visceral layers) and the intestinal mesenteries.
- Identify and describe the regions of the stomach.
- Identify and describe the major sphincters of the gastrointestinal system in relation to their associated structures (oesophageal sphincter, cardiac sphincter, pyloric sphincter, ileocaecal sphincter, hepato‐pancreatic sphincter, anal sphincters).
- Identify and describe the constituent parts of the small intestine (duodenum, jejunum and ileum) and the large intestine (caecum, ascending colon, transverse colon, descending colon, sigmoid colon, rectum and anus).
- Compare and contrast the composition of the walls of the small and large intestines.
- Identify and describe the lobes and major ligaments of the liver, the anatomy of the gallbladder, the anatomy of the pancreas and its associated ducts, and their position relative to the intestines.
- Identify the four quadrants and nine descriptive regions of the abdomen in relation to underlying organs.

### Urinary system

- Identify and describe the main differences between the male and female urinary systems.
- Describe the position of the kidneys and adrenal glands in relation to adjacent structures.
- Identify and describe the external and internal structure of the kidney and the relationship to the associated structures.
- Describe the position of the bladder relative to associated structures in males and females (including during pregnancy).

### Reproductive system

- Identify and describe the differences between the male and female reproductive systems (organs, glands, external genitalia and pelvic characteristics).
- Describe the anatomy of the pelvic diaphragm and perineum and their relationship to the neurovascular structures that supply these regions in males and females.
- Describe the structure and composition of the breast.
- Describe the anatomical changes that occur during pregnancy.

### Lymphatic system

- Describe the drainage of lymph throughout the body.
- Identify the primary (bone marrow & thymus) and secondary lymphoid (lymph nodes, spleen, tonsils and appendix) organs and tissues of the lymphatic system.

### Endocrine

- Identify the major endocrine structures (hypothalamus, anterior and posterior lobes of the pituitary gland, pineal gland thyroid gland and parathyroid glands, thymus, adrenal gland, pancreas, gonads, skin, heart, kidneys, gastrointestinal tract and liver).
